# Effect and safety of traditional Chinese exercises (Qigong therapy) for patients with chronic obstructive pulmonary disease: A protocol for systematic review and meta-analysis

**DOI:** 10.1097/MD.0000000000032284

**Published:** 2022-12-09

**Authors:** Qiumei Zhang, Rongzhen Pang, Zhanhao Cai

**Affiliations:** a Physical Education and Health School of Guangzhou University of Chinese Medicine, China.

**Keywords:** chronic obstructive pulmonary disease, network meta-analysis, Qigong therapy, systematic review

## Abstract

Traditional Chinese Exercises (Qigong therapy) are more and more popular for chronic pulmonary obstructive disease (COPD) patients for the treatment and rehabilitation, however, the comparative effectiveness and safety remains unclear. Our study aims to compare the pros and cons of these exercise interventions for COPD by implementing a network meta-analysis.

**Methods:**

Publicly available scientific databases including ClinicalTrials.gov database, PubMed, Embase database, database in Web of Science, database in Cochrane Library, China Biomedical Literature Service System (SinoMed), Chinese Periodical Service Platform (VIP), China National Knowledge Infrastructure (CNKI), and Wanfang Data Knowledge Service Platform (Wanfang) will be searched for the relevant clinical studies from 2000 to 2022. Randomized controlled trials related to Qigong therapy in COPD treatment will be included. Systematic review and meta-analysis in the current study will be investigated according to the guidelines of the preferred reporting items for systematic reviews and meta-analyses extension statement for reporting of systematic reviews incorporating meta-analyses of health care interventions. The grading of recommendations assessment, development, and evaluation system will be used to evaluate the rank of evidence.

**Results:**

This systematic review will summarize the evidence for different kinds of Qigong therapies.

**Conclusion:**

The network meta-analysis was designed to update and expand on previous research results of clinical trials to better evaluate the effectiveness and safety of different interventions of traditional Chinese exercises for COPD.

## 1. Introduction

Chronic obstructive pulmonary disease (COPD) is a syndrome of progressive airflow limitation resulting in breathlessness and limitations in physical function, often caused by significant exposure to harmful particles or gases and/or alveolar abnormalities,^[1]^ and is becoming a major cause of morbidity and mortality both in China and worldwide.^[2]^ COPD can be divided into acute exacerbation period and stable period. Acute exacerbation of chronic obstructive pulmonary disease (AECOPD) refers to the continuous deterioration of the patient’s respiratory symptoms beyond the daily variation range, and the need to change the drug treatment regimen.^[3]^ AECOPD patients have worsening symptoms and worsening lung function. It takes several weeks to recover. It is also related to the increase in mortality of hospitalized patients is becoming the main cause of complications and death in COPD patients.^[4]^

Exercise training, an important part of pulmonary rehabilitation, such as Tai Chi and Baduanjin, has been shown to improve dyspnea and health status and decrease health care use.^[5]^ Active mind-body movement therapies as an adjunct to or in comparison with pulmonary rehabilitation for people with COPD.^[6]^ Tai Chi is a traditional Chinese mind-body exercise that has been widely practiced in China for many centuries. This exercise has also been applied as a training modality in pulmonary rehabilitation programs for stable COPD.^[7]^ Other excercises like Baduanjin, Yijinjing, Lizijue, and Wuqinxi have similar therapeutic effects.

However, several different traditional Chinese medicine exercises are often used to treat patients with stable COPD. There are many randomized controlled trials (RCTs) and related systematic reviews and meta-analysis to evaluate the efficacy of various traditional Chinese exercise therapies on COPD pulmonary rehabilitation.^[8–10]^ However, most of these studies are designed to compare with conventional Western medical treatment. Few studies compare the effects of different exercise methods. Therefore, neither clinicians nor COPD patients can determine the best option for exercise therapy.

Systematic reviews and meta-analyses are fundamental tools for the generation of reliable summaries of health care information for clinicians, decision makers, and patients.^[11]^ Meta-analyses have usually compared only 2 interventions at a time, but the need to summarize a comprehensive and coherent set of comparisons based on all of the available evidence has led more recently to synthesis methods that address multiple interventions and these methods are commonly referred to as network meta-analysis.^[12,13]^ So, by using network meta-analysis, this systematic review aimed to systematize the different exercises used to deliver pulmonary rehabilitation during COPD and explore which ones are the most effective.

## 2. Materials and Methods

### 2.1. Study protocol registration

We received the registration on PROSPERO platform and finally we got the Registration Number: CRD42021282734. What’s more, this study protocol followed the corresponding guidelines: preferred reporting items for systematic reviews and meta-analyses-P^[14]^ and Cochrane Handbook for Systematic Reviews and Meta-Analysis. The population intervention comparators outcomes study design framework principles were used in the current study.

### 2.2. Clinical study selection

1. Randomized controlled trials of Qigong therapy intervention in COPD patients will be concluded in our systematic review and network meta-analysis.
2. The subjects have diagnosed by authoritative or recognized COPD diagnostic criteria such as the GOLD,^[3]^ GOLD works with health care professionals and public health officials to raise awareness of COPD and to improve prevention and treatment of this lung disease for patients around the world. We focused on stable period patients, the age and disease course are not limited.
3. The observation groups received Qigong therapy (including Taijiquan, Wuqinxi, Ba Duan Jin, Relaxing Gong, Liu Zi Jue) or Qigong therapy combined with conventional treatment, the control group received conventional treatment or no treatment. The control group is composed of participants who do not receive the experimental treatment.
4. Outcome measures included pulmonary function^[15]^: FEV_1_, FEV_1_%, FEV_1_/FVC%; the incidence of acute exacerbations; 6-minutes walking distance^[16]^ and COPD assessment test.^[17]^ It is required to report at least one of the main outcome indicators listed in the included studies.

### 2.3. Exclusion criteria

1. The raw data are missing.
2. Literature comments, experience presentations of individuals or groups, or case reports on nonpharmacologic therapies in exercise-induced fatigue will be excluded.

### 2.4. Data sources and searches

The literature strategy for RCTs from Cochrane Handbook (https://training.cochrane.org/handbook) was applied in this current study protocol. Publicly available scientific databases including ClinicalTrials.gov database (https://clinicaltrials.gov/), PubMed (https://pubmed.ncbi.nlm.nih.gov/), Embase database (https://www.embase.com/), Web of Science (https://www.webofscience.com/), the Cochrane Library database (https://www.cochranelibrary.com/), SinoMed (http://www.sinomed.ac.cn/), China Science and Technology Journal Database (http://qikan.cqvip.com/), China National Knowledge Infrastructure (https://www.cnki.net/), and Wanfang database (https://www.wanfangdata.com.cn/) will be searched for relevant RCTs from 2000 to 2022. Randomized controlled trials (RCTs) that meeting eligibility will be included. These combined texts will be used to search relevant studies: “Qigong,” “COPD,” “randomized controlled trials,” “RCT” “Traditional Chinese Medicine therapies.” Manual searches using the study identifier or references of each study will be also conducted.

### 2.5. Data extraction

Two researchers will perform a duplicate check on the retrieved studies, a preliminary screening of titles and abstracts, and further screening by reading the full text. Data extraction adopts a 2-person entry and cross-checking method. In case of disagreement, we will resolve this through discussion between our researchers. The data extraction content will include title, author, disease diagnosis criteria, disease stage, number of patients, average age, gender, research type, intervention measures, course of treatment, outcomes, follow-up, statistical results, and adverse events.

### 2.6. Quality assessment

The bias risk assessment will be completed independently by 2 investigators using Review Manager (Rev Man, version 5.3) software based on the criteria of the Cochrane Risk of Bias Tool (Cochrane Handbook for Systematic Reviews of Interventions, version 5.1.0).^[18]^ When there is a discrepancy, it will also be resolved through negotiation or by a third investigator. The quality assessment of the included RCTs focused on the essential information from Cochrane Handbook. We will also assess the evidence through grading of recommendations assessment, development, and evaluation system.^[19]^

### 2.7. Statistical analysis

Categorical variable and continuous variable outcomes are respectively used relative risk, mean differenceto represent the effect index, and calculate the 95% confidence interval, if there is a 3-arm test, it will be split into a 2-arm test. Stata/SE 15.0 will be used for the heterogeneity test. If I^2^ ≤ 50% and *P* ≥ .05, it indicates that there is no significant statistical heterogeneity. Meta-analysis can be used to combine the effect size, if I^2^ > 50%, *P* < .05, it indicates there is significant statistical heterogeneity, and then the Meta-regression, subgroup analysis, and sensitivity analysis will be used to explore the source of heterogeneity.^[20]^ We will use Stata/SE 15.0 to make a network meta-analysis evidence relationship diagram, compare the relationship between the interventions, and use the network plot command to draw the evidence network diagram for the comparison of various treatment measures. If there is a line between the points in the figure, it indicates that the 2 Interventions have direct comparison evidence, and no connection indicates that there is no direct comparison evidence. Then we will perform the heterogeneity test and inconsistency test. When there is a closed-loop, the consistency of direct comparison and indirect comparison is judged by the inconsistency factor. When the 95% CI of inconsistency factor starting point is 0, the direct evidence and the indirect evidence is consistent.^[21]^ Then the network package of the Stata software will be used to perform a network Meta-analysis, and the results will be sorted, and the surface under the cumulative ranking curve (SUCRA) of each intervention is calculated. SUCRA is a numeric presentation of the overall ranking and presents a single number associated with each treatment. SUCRA values range from 0 to 100%. The larger the value, the better the therapeutic effect. Finally, draw “comparison-correction” funnel plots to determine whether the research has a small sample research effect.^[22]^

### 2.8. Ethics and dissemination

There is no demand for ethical approval on account of extracted data from published literature, which does not involve patient privacy.

## 3. Discussion

There have been evaluations of the efficacy and safety of traditional fitness exercises on the rehabilitation of COPD patients. It has been found that traditional fitness exercises can improve dyspnea, lung function, exercise endurance, and life quality in patients with stable COPD compared with conventional treatment methods. However, a lack of direct comparison results of different exercise therapies, which limits the clinical application of exercise therapy in stable COPD patients. Perhaps due to the poor quality of the RCT methodology, no definite conclusion can be drawn, and there is still a lack of strong evidence to recommend the best-ranked Qigong therapy.

TaiChi, Baduanjin and Wuqinxi improved physical function in the treatment of COPD and other chronic diseases. TaiChi is beneficial with respect to physical performance, lung function, remission of dyspnea, and quality of life in patients with COPD.^[5,23]^ Wuqinxi is one of the most widely practiced forms of Qigong, it has been used to improve physical and psychological health for thousands of years. By mimicking the postures, movements, and bearing of the animals, along with their corresponding breath adjustment, practitioners will experience an activation of the body, so it is suitable for patients with chronic diseases.^[24,25]^ Liuzijue Qigong is featured by diaphragmatic breathing and pursed-lip breathing can make COPD patients breathing rate slowly and prevent the premature airway occlusion caused by rapid airflow to improve the abnormal breathing pattern of patients.^[26]^ It is helpful for the function of the auxiliary breathing muscles and enhances the flexibility, coordination, and control capacity on neuromuscular limbs to further improve exercise capacity in patients with COPD.^[27]^

## Author contributions

**Conceptualization:** Qiumei Zhang.

**Data curation:** Qiumei Zhang, Rongzhen Pang, Zhanhao Cai.

**Funding acquisition:** Qiumei Zhang.

**Investigation:** Qiumei Zhang.

**Methodology:** Zhanhao Cai, Qiumei Zhang, Rongzhen Pang.

**Project administration:** Qiumei Zhang.

**Software:** Qiumei Zhang, Rongzhen Pang.

**Supervision:** Qiumei Zhang.

**Validation:** Zhanhao Cai.

**Writing** – **original draft:** Qiumei Zhang, Rongzhen Pang, Zhanhao Cai.

**Writing** – **review & editing:** Qiumei Zhang.
